# TRIM2 suppresses prostate cancer progression by promoting ubiquitin-mediated degradation of DTNBP1

**DOI:** 10.3389/fmolb.2026.1945596

**Published:** 2026-09-14

**Authors:** Jiang He, Shen Liu, Wei Tong, Xiao Xiao, Maolin Xiao

**Affiliations:** 1 Department of Urology, University-Town Hospital of Chongqing Medical University, Chongqing, China; 2 Department of Urology, The People’s Hospital of Yubei District of Chongqing City, Chongqing, China; 3 Department of Urology, Chongqing Academy of Medical Sciences, Chongqing General Hospital, Chongqing University, Chongqing, China; 4 Department of Urology, The First Affiliated Hospital of Chongqing Medical University, Chongqing, China

**Keywords:** advanced prostate cancer, DTNBP1, molecular targeting, TRIM2, ubiquitination

## Abstract

**Objective/Background:**

Prostate cancer is the most commonly diagnosed malignancy in men worldwide, with high mortality rates and frequent metastasis at initial diagnosis, underscoring the need to elucidate its molecular mechanisms. TRIM2, an E3 ubiquitin ligase, plays critical roles in several cancers, but its function in prostate cancer remains unclear. This study aimed to investigate the expression, biological function, and clinical significance of TRIM2 in PCa progression.

**Methods:**

TRIM2 expression was analyzed using TCGA, GTEx, and GEO datasets, and validated in PCa and BPH tissues via qRT-PCR, western blot, and immunohistochemistry. Functional assays (CCK-8, wound healing, Transwell, flow cytometry) were performed in DU145 and PC-3 cells with TRIM2 overexpression or knockdown, and xenograft models assessed tumorigenicity in vivo. Proteomic sequencing combined with database prediction (UbiBrowser, BioGRID) identified DTNBP1 as a downstream substrate, confirmed by co-immunoprecipitation, ubiquitination assays, and cycloheximide chase. Drug sensitivity was evaluated using the GDSC database and molecular docking.

**Results:**

TRIM2 was significantly downregulated in PCa tissues, with the lowest levels observed in advanced and castration-resistant cases, and its reduced expression independently predicted worse progression-free interval. ROC analysis demonstrated that TRIM2 effectively discriminated localized from advanced/CRPC disease. TRIM2 overexpression significantly suppressed malignant behaviors *in vivo* and *vitro*. Mechanistically, TRIM2 directly interacted with DTNBP1 and promoted its ubiquitin-proteasome-mediated degradation. Drug sensitivity analysis and molecular docking revealed that TRIM2 expression serves as a predictor of PCa cell sensitivity to clinically relevant agents, including olaparib and navitoclax.

**Conclusion:**

Our findings establish the TRIM2-DTNBP1 axis as a key driver of PCa progression and position TRIM2 as a promising prognostic biomarker and a potential predictive indicator for guiding molecularly targeted therapies in advanced PCa.

## 1 Introduction

Prostate cancer (PCa) consistently exhibits one of the highest diagnosis rates among all male malignant tumors worldwide. Global epidemiological data reveal approximately 1.5 million new cases annually (Bray et al., 2024). According to projections, the annual incidence of PCa is expected to increase from 1.4 million in 2020 to 2.9 million by 2040, with annual deaths increasing from 375,000 to 700,000 (James et al., 2024). Advances in diagnostic techniques and the widespread adoption of early screening have led to a rising number of prostate cancer diagnoses. However, a significant proportion of patients are still initially diagnosed with metastatic disease, thereby losing the opportunity for curative surgery. Moreover, many patients eventually develop castration-resistant prostate cancer (CRPC) after primary therapy (Laccetti and Subudhi, 2017). Consequently, this clinical reality requires us to identify reliable prognostic biomarkers and develop new molecularly targeted therapies.

TRIM2 is a member of the tripartite motif (TRIM) family, which contains over 80 proteins predominantly exhibiting E3 ubiquitin ligase activity (Ozato et al., 2008). TRIM2 drives protein ubiquitination via its RING domain (Balastik et al., 2008; Ohkawa et al., 2001). Previous studies have shown that loss of TRIM2 reduces ubiquitination of the neurofilament light subunit (NF-L), contributing to neurodegenerative disorders (Balastik et al., 2008). Additionally, TRIM2 exerts a neuroprotective function during rapid ischemic tolerance by mediating the ubiquitination and subsequent downregulation of the pro-apoptotic protein Bcl-2 interacting mediator of cell death (Bim), as well as by modulating the p42/p44 MAPK-dependent ubiquitination pathway (Thompson et al., 2011).

In cancer research, TRIM2 expression is controlled by the GPER–MAPK/ERK pathway in breast cancer, thereby facilitating ubiquitin-proteasome-dependent degradation of Bim (Yin et al., 2017). Another study reported TRIM2 promotes M2 polarization of tumor-associated macrophages by targeting IκBα for ubiquitination and degradation in colon cancer cells, thereby triggering NF-κB pathway activation (Liu Z. et al., 2024). Furthermore, in pancreatic ductal adenocarcinoma, TRIM2 upregulates NRF2, mediating reactive oxygen species (ROS) metabolic reprogramming and activating the NRF2/ITGB7/FAK signaling axis to enhance malignant phenotypes (Sun et al., 2020). However, its expression profile, biological function, and clinical significance in PCa, particularly in the context of advanced disease, remain largely unexplored.

Here, we assessed TRIM2’s role in PCa progression, its prognostic significance, and its potential as a therapeutic target. We demonstrate that TRIM2 is significantly downregulated in PCa, with its lowest expression observed in advanced and CRPC stages, and that its loss is an independent predictor of poor prognosis. Experiments demonstrated that TRIM2 suppressed prostate cancer cell migration, invasion and proliferation, yet facilitated apoptosis. We identified DTNBP1 as a novel downstream effector of TRIM2, which is subjected to ubiquitin-proteasome-mediated degradation. Finally, we provide evidence that TRIM2 expression correlates with PCa cell sensitivity to PARP and BCL-2 inhibitors. Our findings not only reveal a novel tumor-suppressive mechanism but also highlight TRIM2 as a promising prognostic biomarker and a potential predictive tool for guiding molecularly targeted therapy in advanced PCa.

## 2 Materials and methods

### 2.1 TCGA, GEO, and CCLE data processing and analysis

Transcriptomic profiles and clinicopathological data of prostate adenocarcinoma were retrieved from The Cancer Genome Atlas (TCGA) via the UCSC Xena platform (https://xenabrowser.net/), while normal prostate gene expression data came from the Genotype-Tissue Expression (GTEx) database (https://www.gtexportal.org/home/), and the expression levels of TRIM2 in each sample were log2-transformed prior to subsequent analyses. We further validated the expression pattern of TRIM2 in PCa using three Gene Expression Omnibus (GEO) (https://www.ncbi.nlm. nih. gov/geo/) datasets: GSE32751 (Kuner et al., 2013), GSE71016 (Zhang et al., 2016) and GSE80609 (Yun et al., 2017). Based on the Cancer Cell Line Encyclopedia (CCLE) database (https://sites.broadinstitute. org/ccle), Baseline TRIM2 expression in prostate cancer cell lines was further quantified.

### 2.2 Diagnosis and prognosis analysis

The association between TRIM2 expression and clinical outcomes in PCa patients was assessed using receiver operating characteristic (ROC) curve and survival analyses utilizing the TCGA-PRAD cohort. The R packages “pROC” and “ggplot2” are used for ROC analysis and visualization. Overall survival (OS), progression-free interval (PFI), disease-free interval (DFI), and disease-specific survival (DSS) data were extracted for each sample of TCGA from previous studies (Liu et al., 2018). Patients were dichotomized into high- and low-TRIM2 expression groups based on an optimal cutoff derived from the ‘maxstat’ R package, with the constraint that each group constituted a minimum of 25% and a maximum of 75% of the total cohort. The resulting optimal cutoff value was 2.3564. Subsequently, the ‘survfit’ function in the ‘survival’ package was employed to plot Kaplan-Meier (KM) survival curves, followed by the log-rank test for intergroup comparisons.

### 2.3 Enrichment analysis

To systematically explore the biological functions of TRIM2 in PCa, we conducted an analysis using a series of bioinformatics approaches. Based on the median expression level, all samples were categorized into TRIM2-high and TRIM2-low groups. The R package “limma” was then applied for differential expression analysis, identifying differentially expressed genes (DEGs) as those satisfying false discovery rate (FDR) < 0.05 and |log2 fold change| > 1.5. The “clusterProfiler” R package was used to conduct Gene Ontology (GO) (Ashburner et al., 2000) and Kyoto Encyclopedia of Genes and Genomes (KEGG) (Kanehisa and Goto, 2000) pathway enrichment analyses on the DEGs, with GO annotation covering molecular function (MF), biological process (BP), and cellular component (CC) (The Gene Ontology, 2019), providing a comprehensive functional characterization of the DEGs. Subsequently, gene set enrichment analysis (GSEA) (Subramanian et al., 2005) was conducted with the same package, employing the curated (c2. cp.all.v2022.1. Hs.symbols.gmt) and Hallmark (h.all.v2022.1. Hs.symbols) gene sets from MSigDB.

### 2.4 Drug sensitivity analysis and molecular docking

We systematically assessed the effect of TRIM2 expression on drug sensitivity using data from the Genomics of Drug Sensitivity in Cancer (GDSC) database (https://www.cancerrxgene.org/) (Yang et al., 2013). Using the ‘pRRophetic’ R package and a ridge regression model, the half-maximal inhibitory concentration (IC50) value was determined to represent drug sensitivity. A higher IC50 value indicates greater cellular resistance. Drug sensitivity differences based on TRIM2 expression levels were assessed, and Spearman’s rank correlation test was performed to correlate TRIM2 expression with drug IC50 values. To further predict the potential interaction between TRIM2 and existing therapeutic agents for prostate cancer, molecular docking studies were conducted. The selected drugs included bicalutamide, docetaxel, olaparib, and the BCL-2 inhibitor navitoclax. Molecular docking was conducted using AutoDock Vina 1.2.7 (Eberhardt et al., 2021; Trott and Olson, 2010). The TRIM2 structure was preprocessed in PyMOL 3.0.3 via removal of water and non-essential ligands and addition of hydrogens. The small molecule structures of each drug were energy-minimized and optimized using ChemDraw 20.0. Subsequently, PDBQT files for both the protein and ligands were generated using AutoDock Tools 1.57. A docking grid box covering the entire protein was defined to allow a global search. Finally, Ultimately, PyMOL 3.0.3 and Discovery Studio Visualizer were used to visualize and analyze the predicted binding sites and interaction modes. Although bioinformatic predictions do not directly equate to actual *in vivo* therapeutic efficacy, the GDSC-derived IC50 estimations, based on high-throughput pharmacogenomic screening, provide a reliable and reproducible reference for identifying potential drug-gene correlations and generating clinically testable hypotheses.

### 2.5 Tissue acquisition and specimen preparation

A total of 30 prostate cancer and 30 benign prostatic hyperplasia (BPH) specimens were obtained from the Department of Urology, Chongqing General Hospital. Written informed consent was secured from all participants, and the study received ethical approval from the Clinical Research Ethics Committee (Ethics Approval No.: IIT S2026-018–01). Fresh tissues were split into two portions: one snap-frozen in liquid nitrogen for RNA/protein isolation, and the other fixed in 10% neutral buffered formalin for paraffin, followed by paraffin embedding and sectioning for further analysis.

### 2.6 RNA extraction and quantitative real-time PCR

Liquid nitrogen-frozen PCa and BPH tissues were used for RNA extraction via a commercial kit (ABclone, China). RNA purity (A260/A280 ratio) and concentration were determined by microspectrophotometry, and qualified samples were stored at −80 °C. cDNA was reverse-transcribed using the ABScript II Kit (ABclone, China). qPCR was performed on a Bio-Rad system with SYBR Green Mix. A standard curve was included in each run to assess primer amplification efficiency. Each sample was run in triplicate. Primer sequences were: TRIM2: Forward: 5‘-CAGGTTTGGTAGCCGAGGAAATG-3’, Reverse: 5‘-CTGCCCATTTCCTTCTCCATTTG-3’; GAPDH: Forward: 5‘-GGAGTCCACTGGCGTCTTCA-3’, Reverse: 5‘-GTCATGAGTCCTTCCACGATACC-3’. RNA extraction from cultured cells followed an identical procedure to that described for tissue samples, in strict accordance with the respective kit’s protocol.

### 2.7 Protein extraction and Western blot

An identical protein extraction protocol was applied to both tissue and cell samples. Briefly, tissues or cell pellets were placed in EP tubes and subjected to ice-cold lysis for 30 min using a protein lysis buffer (RIPA:PMSF = 1:1). The lysates were then centrifuged to recover the protein supernatant, whose concentrations were measured via BCA assay. Equivalent protein amounts were loaded and separated by SDS-PAGE using gels cast specifically for the molecular weights of the proteins of interest. After separation, proteins were electrotransferred to PVDF membranes, which were subsequently blocked with 5% skim milk. Primary antibody incubation was carried out overnight at 4 °C, and secondary antibodies linked to HRP were incubated for 2 h at room temperature. Finally, protein bands were detected with an ECL substrate (PromoCell, Germany) and captured with a BIO-RAD chemiluminescence imaging system. The antibodies used were as follows: anti-TRIM2 (rabbit, ab3942, Abcam), anti-DTNBP1 (mouse, sc-390626, Santa Cruz Biotechnology), anti-Ki67 (rabbit, 27309-1-AP, Proteintech), anti-Bim (rabbit, 22037-1-AP, Proteintech), anti-Caspase-3 (rabbit, ab32351, Abcam), anti-GAPDH (rabbit, 10494-1-AP, Proteintech), and anti-β-Actin (mouse, 4,968, Cell Signaling Technology).

### 2.8 Hematoxylin-eosin staining (HE) and immunohistochemistry (IHC)

Tissue sections were heat-fixed at 55 °C for 3 h to enhance their attachment to slides. Deparaffinization was performed in xylene, followed by rehydration via a descending ethanol gradient (100%→70%, 5 min each). Antigen retrieval was conducted in preheated sodium citrate buffer (15 min, pH 6.0, >95 °C). After blocking endogenous peroxidase with 3% H_2_O_2_(15 min, room temperature), ssections were incubated overnight at 4 °C with primary antibody (1:400) and subsequently for 20 min at 37 °C with HRP-conjugated secondary antibody. Color was generated with diaminobenzidine (DAB) under light protection, then hematoxylin counterstaining was carried out. Finally, after dehydration and clearing, the sections were mounted with neutral resin. Light microscopy was used for observation and imaging. For HE, following the same deparaffinization and rehydration steps as described for IHC, sections were stained with hematoxylin (pH 7.0, 20 min, dark, room temperature), differentiated in 1% acid alcohol, and blued in running water. Cytoplasmic staining was achieved with 0.5% eosin alcohol solution for 3 min. After rinsing, dehydration, clearing, and mounting were carried out according to the IHC procedure.

### 2.9 Cell lines, culture and cell transfection

DU 145, LNCaP, and PC-3) used in this study were obtained from the Cell Bank of the Shanghai Institute of Life Sciences, Chinese Academy of Sciences. Cell lines were grown respectively in high-glucose DMEM, RPMI 1640, and F12K medium (Gibco, United States), all supplemented with 1% penicillin-streptomycin (Invitrogen, United States) and 10% fetal bovine serum (FBS) (Multicell, United States); culture conditions were 37 °C in a humidified 5% CO_2_ incubator. Cells were cultured to the logarithmic phase for transfection, then 2 × 10^5^ cells/well were seeded into 6-well plates, and allowed to attach until reaching 25%–30% confluence prior to transfection. Lentiviral constructs for TRIM2 overexpression, knockdown, and corresponding controls were purchased from GeneChem (Shanghai, China). The multiplicity of infection (MOI) was used to calculate the volume of lentivirus, and all subsequent transfection steps were performed exactly as specified by the manufacturer. Cell lines with stable transfection were created and utilized for later experiments. All cell-based functional assays (including CCK-8, wound healing, Transwell, and flow cytometry) were performed three independent biological replicates (n = 3 per group), with three technical replicates per experiment.

### 2.10 Proteomics sequencing and protein interaction prediction

To identify potential interacting partners and ubiquitination substrates of TRIM2, we combined proteomic sequencing with queries of publicly available interaction databases. For proteomic analysis, we performed quantitative proteomic analysis using DU145 cells stably overexpressing TRIM2 and empty vector control cells, with three biological replicates per group. Cells were lysed directly in the dishes with TRIzol® reagent once they reached the logarithmic growth phase. The lysates were transferred to pre-cooled cryovials, rapidly frozen in liquid nitrogen, and stored on dry ice. Samples were sent to the BGI DNBSEQ platform for analysis (BGI Genomics, Shenzhen, China). To uncover TRIM2-interacting protein candidates from the proteomic data, we adopted an intersection-based filtering strategy rather than relying solely on a fold-change cut-off (e.g., |log2FC| > 1.5). Specifically, we cross-referenced the entire set of identified proteins with two independent resources (Bray et al., 2024): the E3 ligase-substrate interaction predictions from the UbiBrowser 2.0 platform (using the “E3 ubiquitin ligase” prediction module) (http://ubibrowser.ncpsb.org/) (Li et al., 2017) and the experimentally verified physical interactors of TRIM2 retrieved from the BioGRID database (https://thebiogrid.org/). This approach was designed to prioritize functionally relevant and high-confidence candidates by excluding proteins lacking prior evidence of E3 interaction networks. The intersecting candidates were visualized using the R packages “VennDiagram” and “ggplot2”2.11 CCK-8 Assay.

Single-cell suspensions of stably transfected cells were seeded into 96-well plates at 2,000 cells per well (100 µL medium, five replicates per group). A CCK-8 working solution (100 μL; complete medium:CCK-8 = 100:1; Promega, China) was added to assess viability at 0, 24, 48, 72, and 96 h. Edge effects were minimized by adding 100 µL PBS to peripheral wells. Incubation was carried out at 37 °C for 2 h in darkness, followed by measurement of absorbance at 450 nm using an ELISA reader (Bio-Rad, United States of America).

### 2.11 Scratch test

Cells in 6-well plates were cultured to >90% confluence. Two straight, parallel scratches were introduced perpendicularly to pre-marked reference lines on the plate bottom using a sterile 10-μL pipette tip. Detached cells were removed by two gentle washes with pre-warmed PBS, afollowed by culture in serum-free medium. The scratches were photographed immediately (0 h) at predetermined locations under a microscope. Following 24 h of incubation, images were captured again at the identical positions.

### 2.12 Transwell assays

Transwell assays were performed using 8-μm pore size inserts (Jet Biofil, Guangzhou, China). Transwell migration was performed by seeding 2 × 10^4^ cells (200 μL, serum-free medium) into upper inserts and adding 600 μL of 10% FBS medium to the lower chamber. Following 24 h, migrated cells were fixed (4% paraformaldehyde, 15 min) and stained (0.1% crystal violet in methanol, 20 min). Cells that did not migrate on the upper side were removed. For invasion, 60 µL of Matrigel, diluted 1:8 with serum-free medium was added to the upper chamber and solidified, followed by the same procedure as migration. Cells on the chamber membrane were visualized using an inverted microscope (three random fields per replicate).

### 2.13 Flow cytometric detection of cell apoptosis

Cells floating in the culture medium were collected, and adherent cells were prepared as a single-cell suspension and combined with the floating cells. After centrifugation and removal of the supernatant, then resuspended pellet in 1 mL of PBS. After a second centrifugation, the pellet was resuspended in 500 µL of PBS and transferred to a 1.5 mL EP tube. After labeling with Annexin V-FITC and propidium iodide (PI) according to the supplier’s protocol (Beyotime, China), cells were kept in the dark for 20 min at room temperature. Finally, stained cells were subsequently measured on a flow cytometer (ThermoFisher, United States).

### 2.14 Immunofluorescence assay

Sterile coverslips placed in 24-well plates were seeded at a density below 5 × 10^3^ cells/well. Following 12 h of culture, cellular fixation was carried out with 4% paraformaldehyde for 30 min, followed by permeabilization using 0.5% Triton X-100. Nonspecific binding was blocked by incubating with 5% BSA for 60 min at room temperature. Overnight incubation at 4 °C was performed with a 1:1 mixture of anti-TRIM2 and anti-DTNBP1 primary antibodies (diluted per manufacturer’s instructions). Following primary antibody removal and washing, the samples were treated with fluorophore-labeled secondary antibody working solution for 1 h at room temperature under dark conditions. DAPI staining (10 min) was used for nuclear counterstaining, followed by mounting with anti-fade medium. After a further 30 min dark incubation, images were acquired with a fluorescence microscope (Leica, Germany).

### 2.15 Animal model

Four-week-old male BALB/cA-nu nude mice (Chongqing Tengxin Biotechnology Co., Ltd.) were randomly split into two groups (n = 5 mice per group). Animal experiments were authorized by the Animal Experiment Ethics Committee of the First Affiliated Hospital of Chongqing Medical University (Approval No. 2021–405). DU145 cells, either overexpressing TRIM2 or as empty vector controls, were resuspended in PBS and injected subcutaneously (2 × 10^7^ cells/mouse) into the right scapular region. The growth of the tumor was tracked every 3–4 days by measuring its longest (length) and shortest (width) diameters with a caliper. Tumor volume was calculated using the formula: volume = length × width^2^/2. At the experimental endpoint, cervical dislocation was performed for euthanasia. Tumors were immediately dissected, excised completely, and then measured for volume and wet weight. A portion of fresh tumor tissue was used for protein extraction, while the remainder is used to prepare paraffin sections.

### 2.16 Co-immunoprecipitation (Co-IP) assay

Cells grown to about 95% confluence in 10 cm dishes were used to extract total proteins. The Input group was prepared by mixing 50 μL of lysate supernatant with an equal volume of 2× SDS loading buffer. The remaining supernatant was divided into two equal groups: one was immunoprecipitated with 2 μg of anti-TRIM2 antibody, and the other served as a control using species-matched non-specific IgG. After overnight rotation at 4 °C, each sample received 40 µL of pre-washed Protein A/G agarose beads, followed by another 3 h rotation at 4 °C. Centrifugation at 1,000 × g for 5 min was used to pellet the beads, which were then washed 4 times with 1 mL of cold IP lysis buffer, and resuspended in 20 μL of 2× SDS loading buffer. Denaturation at 100 °C for 5 min was performed, after which the mixture was given a short centrifugation. Recovered proteins were subjected to Western blot analysis to confirm the interaction of TRIM2 with DTNB1.

### 2.17 Ubiquitination assay

Upon reaching approximately 80% confluence, cells were exposed to 20 μM MG132 for more than 6 h. After washing twice with ice-cold PBS, we lysed the cells on ice for 30 min in 1 mL of chilled immunoprecipitation lysis buffer containing protease inhibitors. Following the collection of the lysate, the protein supernatant (300 µL) was incubated with 1 µg of DTNBP1-specific primary antibody at 4 °C overnight with gentle rotation. Protein A/G magnetic beads were added and rotated for 2 h at 4 °C. Following three washes with cold lysis buffer, then mixed with 30 μL of loading buffer, vortexed, and denature at 100 °C for 10 min. Finally, the ubiquitination status of DTNBP1 was detected by Western blot.

### 2.18 Protein degradation assay

At ∼70% confluence, cells were exposed to 20 μM cycloheximide (CHX) (Aladdin, China) to suppress ongoing protein synthesis. To assess protein stability, cell lysates were prepared at serial time points (0, 6, 12, and 18 h) after CHX treatment. Western blot was then employed to quantify DTNBP1 and TRIM2 levels, allowing calculation of their degradation rates.

### 2.19 Statistical analysis

Differences in TRIM2 expression were evaluated using the independent samples t-test, and survival curves were generated via the Kaplan-Meier method with log-rank testing. Univariate and multivariate Cox regressions assessed associations between TRIM2 expression and prognosis. Continuous variables (mean ± SD) and categorical variables (frequency/percentage) were compared via t-test and chi-square test, respectively. ImageJ software quantified Western blot bands, cell counts, cell migration rate, and integrated optical density (IOD). For data including tumor growth curves and CCK-8 proliferation assays, statistical comparisons between groups were performed using two-way analysis of variance (ANOVA) followed by multiple comparisons. All analyses and plots (curve/bar graphs) were performed using GraphPad Prism 8.0 software and R 4.2.1. Statistical significance was set at *P* < 0.05.

## 3 Results

### 3.1 TRIM2 is significantly downregulated in PCa tissues

To evaluate TRIM2 expression levels in PCa, an integrative analysis was performed using RNA-seq data from TCGA and GTEx databases. TRIM2 was found to be markedly downregulated in PCa versus normal tissues (Figure 1A), including in paired tumor and adjacent normal samples (Figure 1B). This result was corroborated by three independent GEO datasets (GSE32571, GSE71016) (Figures 1C,D). Furthermore, the GSE80609 data demonstrated markedly reduced TRIM2 levels in advanced/CRPC lesions relative to primary tumors (Figure 1E). Clinical PCa samples showed a significant reduction in both TRIM2 mRNA and protein levels (Figures 1F–H). Moreover, a significant association was identified between TRIM2 expression and T stage (P = 0.022), while no statistically significant associations were observed with N stage, Gleason score, or baseline PSA levels, potentially due to the limited sample size (Table 1). These findings suggest that loss of TRIM2 parallels malignant progression and correlates with aggressive, late stage disease. It should be noted that the relatively limited sample size in this study may not fully elucidate the comprehensive role and clinical implications of TRIM2 in PCa.

**FIGURE 1:**
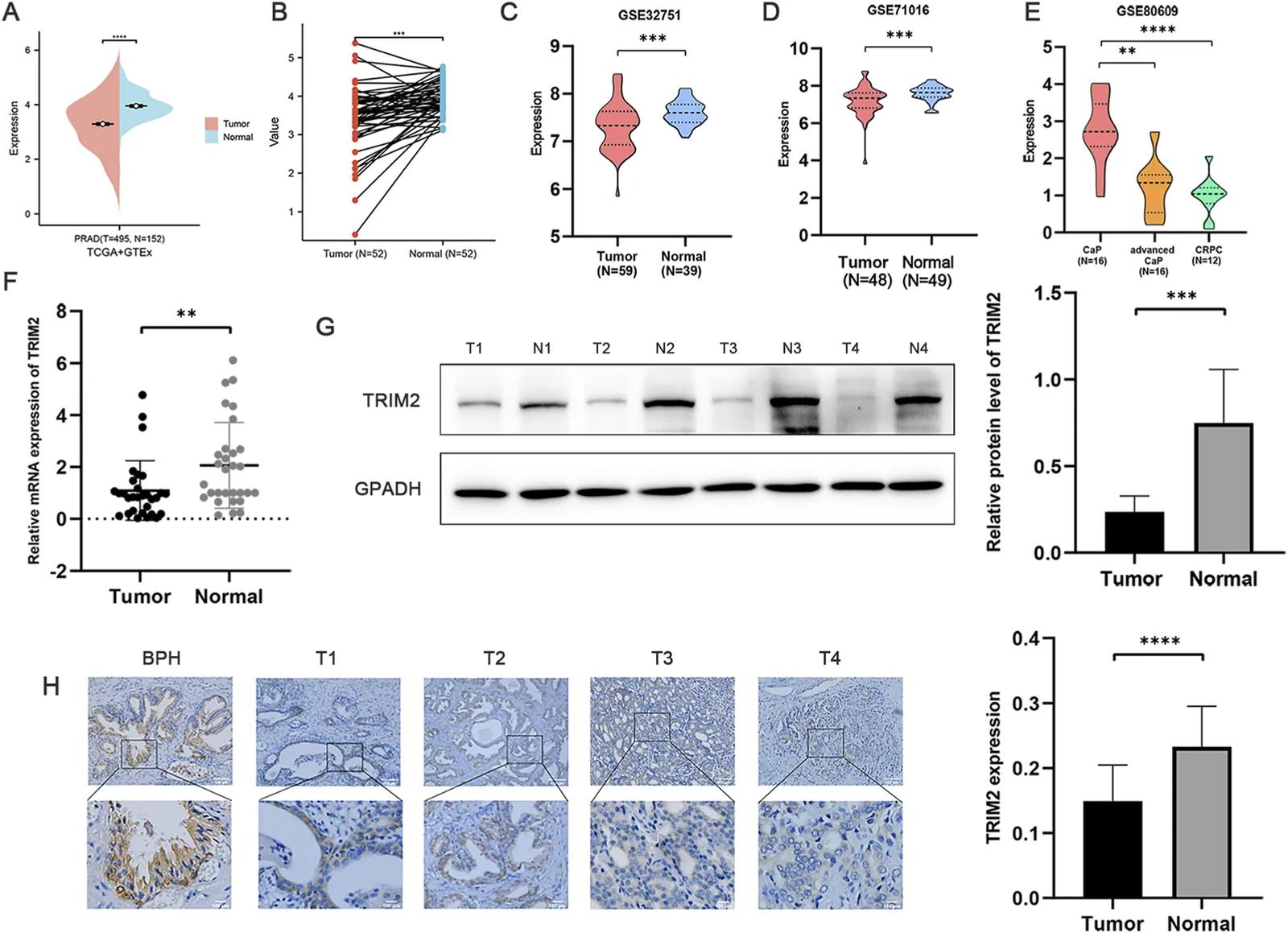
Downregulation of TRIM2 in Prostate Cancer Tissues. **(A)** Combined analysis of TRIM2 expression in PCa using TCGA and GETx databases. **(B)** TRIM2 expression difference between PCa and adjacent normal tissues in TCGA; **(C–E)** GEO datasets (GSE32751, GSE71016 and GSE80609) independently validate TRIM2 expression in PCa. **(F–H)** qRT-PCR, Western blot, and IHC further confirm the downregulation of TRIM2 in PCa. **P < 0.01, ***P < 0.001, ****P < 0.0001.

**TABLE 1:** Relationship between TRIM2 expression and clinicopathologic features of PCa patients.

Characteristics	Low expression	High expression	P value
n	15	15	
T stage, n (%)			0.022
4	5 (16.7%)	0 (0%)	
1	2 (6.7%)	1 (3.3%)	
2	7 (23.3%)	8 (26.7%)	
3	1 (3.3%)	6 (20%)	
N stage, n (%)			0.224
0	15 (50%)	12 (40%)	
1	0 (0%)	3 (10%)	
M stage, n (%)			0.224
0	15 (50%)	12 (40%)	
1	0 (0%)	3 (10%)	
Gleason score, n (%)			0.085
7	7 (23.3%)	3 (10%)	
≥8	8 (26.7%)	8 (26.7%)	
≤6	0 (0%)	4 (13.3%)	
tPSA, n (%)			0.700
<20 ng/ml	10 (33.3%)	9 (30%)	
≥20 ng/ml	4 (13.3%)	6 (20%)	
≥21 ng/ml	1 (3.3%)	0 (0%)	
Age, n (%)			0.100
≥60	15 (50%)	11 (36.7%)	
<60	0 (0%)	4 (13.3%)	

### 3.2 Diagnostic and prognostic significance of TRIM2 in PCa

ROC analysis of TCGA data revealed a diagnostic AUC of 0.787 for TRIM2 in PCa. We validated this finding using three independent GEO datasets (GSE32571, GSE71016). The AUC values of TRIM2 were 0.704 and 0.721 (Figures 2B,C). The GSE80609-based ROC analysis yielded an AUC of 0.923 (95% CI: 0.828–1.000; Figure 2D) for TRIM2 in distinguishing localized PCa from advanced/CRPC, indicating that it has excellent diagnostic performance. This finding validates that declining TRIM2 expression strongly correlates with progression to advanced, castration-resistant phenotypes. In terms of prognosis, KM survival analysis revealed that high TRIM2 expression correlated with significantly better OS, PFI, and DFI, but not with DSS (Figures 2E–H). Univariate Cox regression identified TRIM2 expression, pathological T/N stage, clinical T stage, Gleason score, and serum PSA level as significant correlates of PFI. Multivariate Cox regression analysis revealed that high TRIM2 expression conferred a 39.7% reduction in the risk of disease progression (HR = 0.603, 95% CI: 0.364–0.999, P = 0.0496), independent of conventional clinicopathological factors including Gleason score,T and N stage (Table 2). These findings indicate its potential utility in refining current risk stratification systems.

**FIGURE 2:**
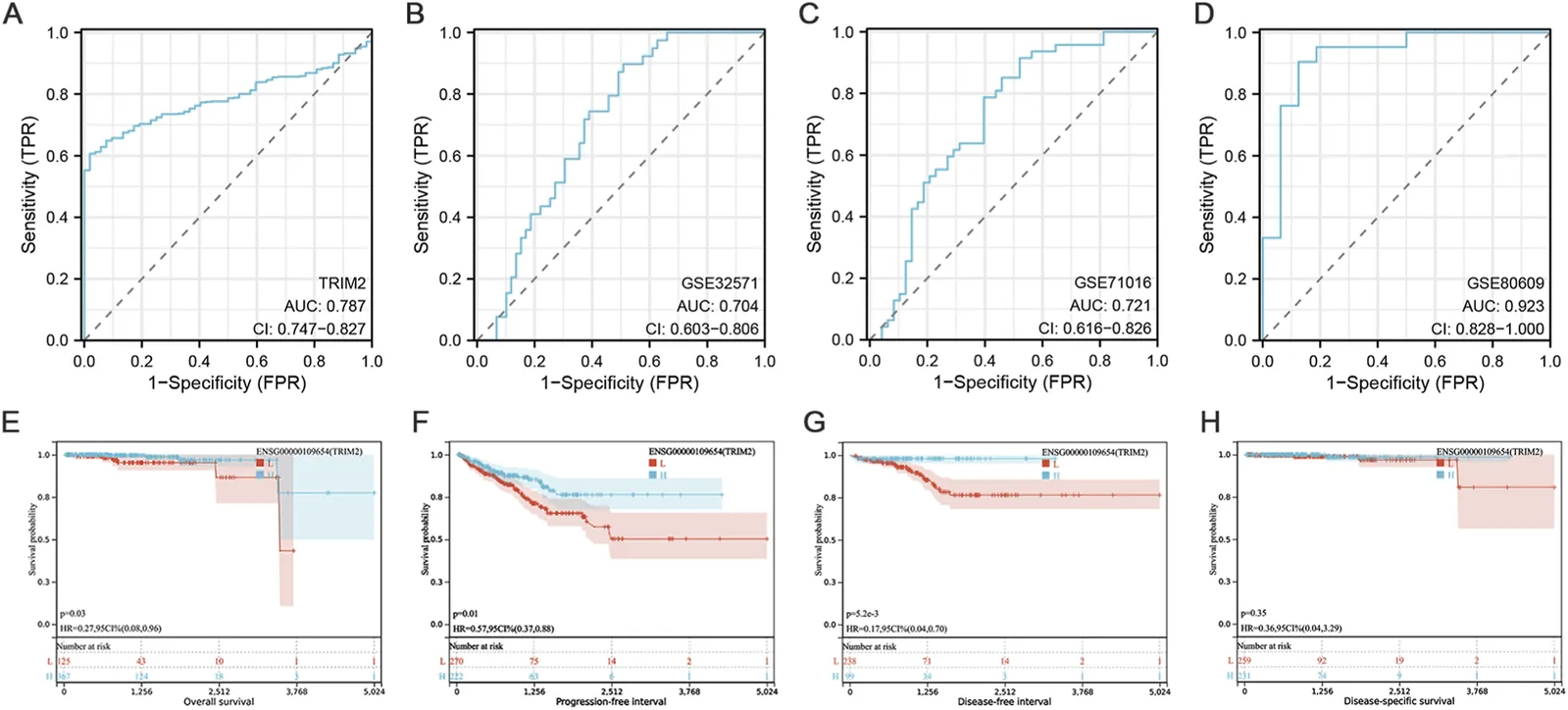
Evaluation of TRIM2 expression for diagnosing and predicting outcomes in PCa. **(A–D)** ROC curves derived from TCGA and GEO datasets evaluating the diagnostic performance of TRIM2. **(E–H)** KM survival analyses showing the associations between TRIM2 expression levels and OS, PFI, DFI, and DSS in PCa patients.

**TABLE 2:** Cox regression analysis using PFI as a prognostic category.

Characteristics	Total(N)	Univariate analysis		Multivariate analysis	
		Hazard ratio (95% CI)	P value	Hazard ratio (95% CI)	P value
TRIM2	501				
Low	250	Reference		Reference	
High	251	0.620 (0.409–0.940)	**0.024**	0.603 (0.364–0.999)	**0.0496**
Pathologic T stage	494				
T2	189	Reference		Reference	
T3&T4	305	3.745 (2.118–6.623)	**5.64e-06**	1.775 (0.840–3.749)	0.1326
Clinical T stage	407				
T1&T2	352	Reference		Reference	
T3&T4	55	3.799 (2.357–6.123)	**4.27e-08**	1.995 (1.157–3.438)	**0.0129**
Pathologic N stage	428				
N0	348	Reference		Reference	
N1	80	1.901 (1.175–3.078)	**0.0089**	1.227 (0.698–2.156)	0.4766
Clinical M stage	460				
M0	457	Reference			
M1	3	3.587 (0.497–25.906)	0.2055		
Gleason score	501				
6	46	Reference		Reference	
7	248	4.362 (0.590–32.245)	0.149	18,097,592.9195 (0.000 - inf)	0.996
8and9and10	207	17.934 (2.490–129.154)	**0.0042**	49128701.1623 (0.000 - inf)	0.996
PSA (ng/mL)	444				
<4	417	Reference		Reference	
≥ 4	27	4.223 (2.108–8.459)	**4.82e-05**	2.530 (1.097–5.837)	**0.0295**
Age	501				
≤ 60	225	Reference			
>60	276	1.304 (0.865–1.967)	0.205		

Bolded values indicate statistical significance.

### 3.3 Validation of TRIM2 overexpression and knockdown efficiency

TRIM2 expression was assessed in PC-3, LNCaP, and DU 145 cell lines. The CCLE database was first analyzed, revealing that TRIM2 expression was lowest in DU 145 cells and highest in LNCaP cells (Figure 3A). However, subsequent qRT-PCR and Western blot assessments uncovered a different pattern: highest in PC-3 cells, intermediate in LNCaP cells, and lowest in DU 145 cells. (Figures 3B,C). This discrepancy may be attributable to several factors. First, CCLE datasets typically represent a basal mRNA abundance under standard reference culture conditions, which can differ substantially from our specific culture protocols (e.g., media composition, serum concentration, and passage number). Second, the phenotypic heterogeneity that accumulate over prolonged passages in laboratories can lead to divergent expression profiles of TRIM2. Consequently, we selected the cell lines for functional experiments (DU145 for overexpression and PC-3 for knockdown) based on our validated expression levels rather than solely on the public database outputs. Lentiviral transduction efficiency was assessed by qRT-PCR, demonstrating that both shTRIM2#1 and shTRIM2#2 sequences exhibited high knockdown efficiency (Figure 3D). We selected the 2 cell lines showing the highest knockdown efficiency, together with the TRIM2-overexpressing cell line, for further cultured, and transfection efficiency was confirmed by Western blot. The results confirmed successful TRIM2 overexpression in DU 145 cells and efficient knockdown in PC-3 cells (Figures 3E,F).

**FIGURE 3:**
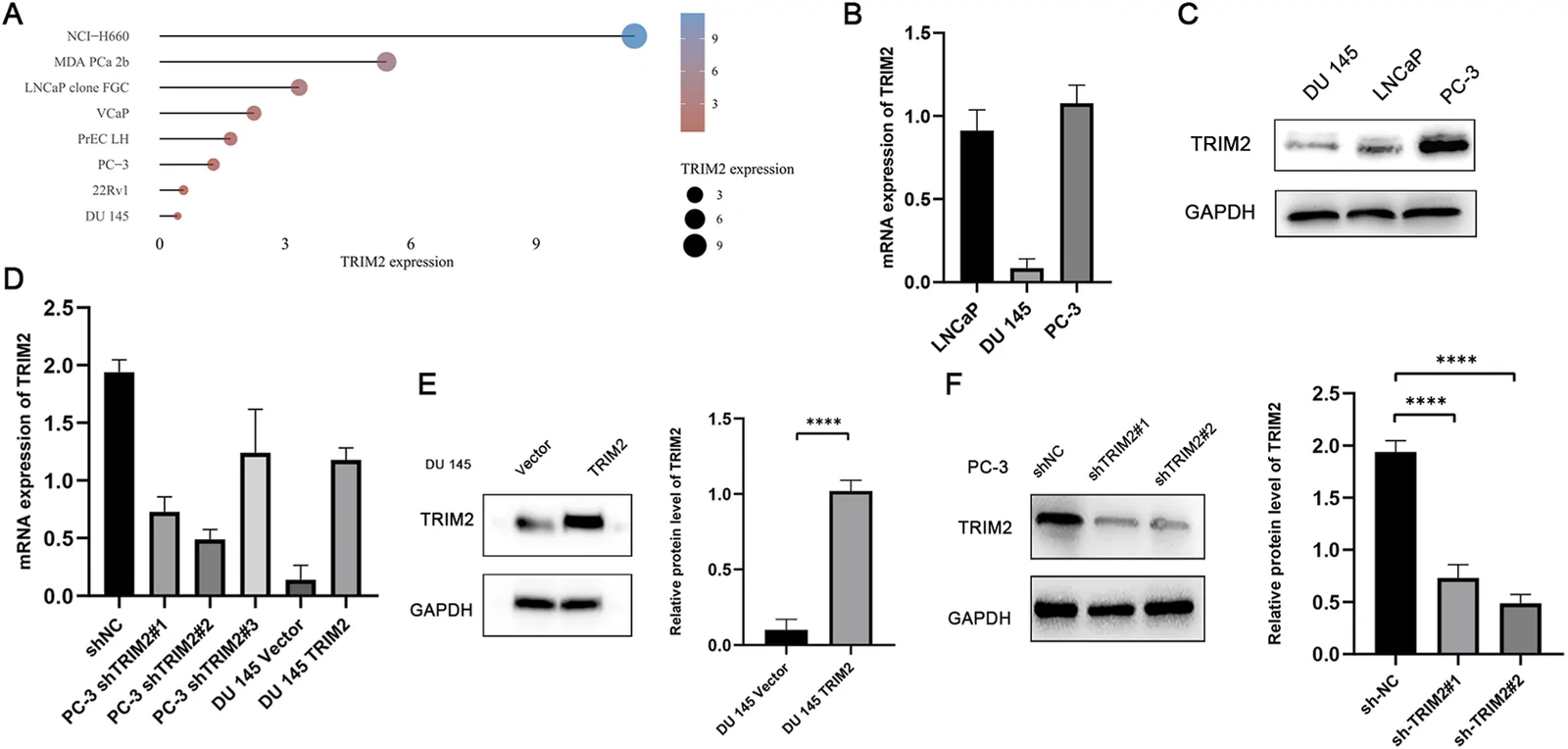
Screening of cell lines and validation of TRIM2 transfection efficiency. **(A)** Baseline TRIM2 expression levels in multiple prostate cancer cell lines were assessed using the CCLE database. **(B–C)** Expression of TRIM2 in PC-3, LNCaP and DU 145 cells was validated by qRT-PCR and Western blot, respectively. **(D)** Overexpression and knockdown efficiencies of TRIM2 via lentiviral were determined by qRT-PCR. **(E–F)** TRIM2 protein alterations following overexpression and knockdown were validated by Western blot. ****P < 0.0001.

### 3.4 TRIM2 regulates malignant behaviors of prostate cancer cells *in Vitro*

Functional assays were performed to assess TRIM2’s regulation of prostate cancer cell malignancy. Wound healing assays indicated that TRIM2 overexpression reduced DU 145 cell migration (Figure 4A), while TRIM2 knockdown promoted PC-3 cell migration (Figure 4B). Transwell assays further confirmed that TRIM2 overexpression substantially inhibited DU 145 cell migration and invasion (Figure 4C), whereas TRIM2 knockdown significantly promoted these phenotypes in PC-3 cells (Figure 4D). Flow cytometry analysis showed that TRIM2 overexpression significantly increased the apoptosis rate in DU 145 cells, whereas TRIM2 knockdown decreased apoptosis in PC-3 cells (Figures 4E,F). CCK-8 assays demonstrated that proliferative activity was suppressed by TRIM2 overexpression in DU 145 cells (Figure 4G), and promoted by TRIM2 knockdown in PC-3 cells compared with negative controls (Figure 4H). Collectively, these data demonstrate that therapeutic restoration of TRIM2 activity could represent a promising strategy to impede prostate cancer progression and manage advanced disease.

**FIGURE 4:**
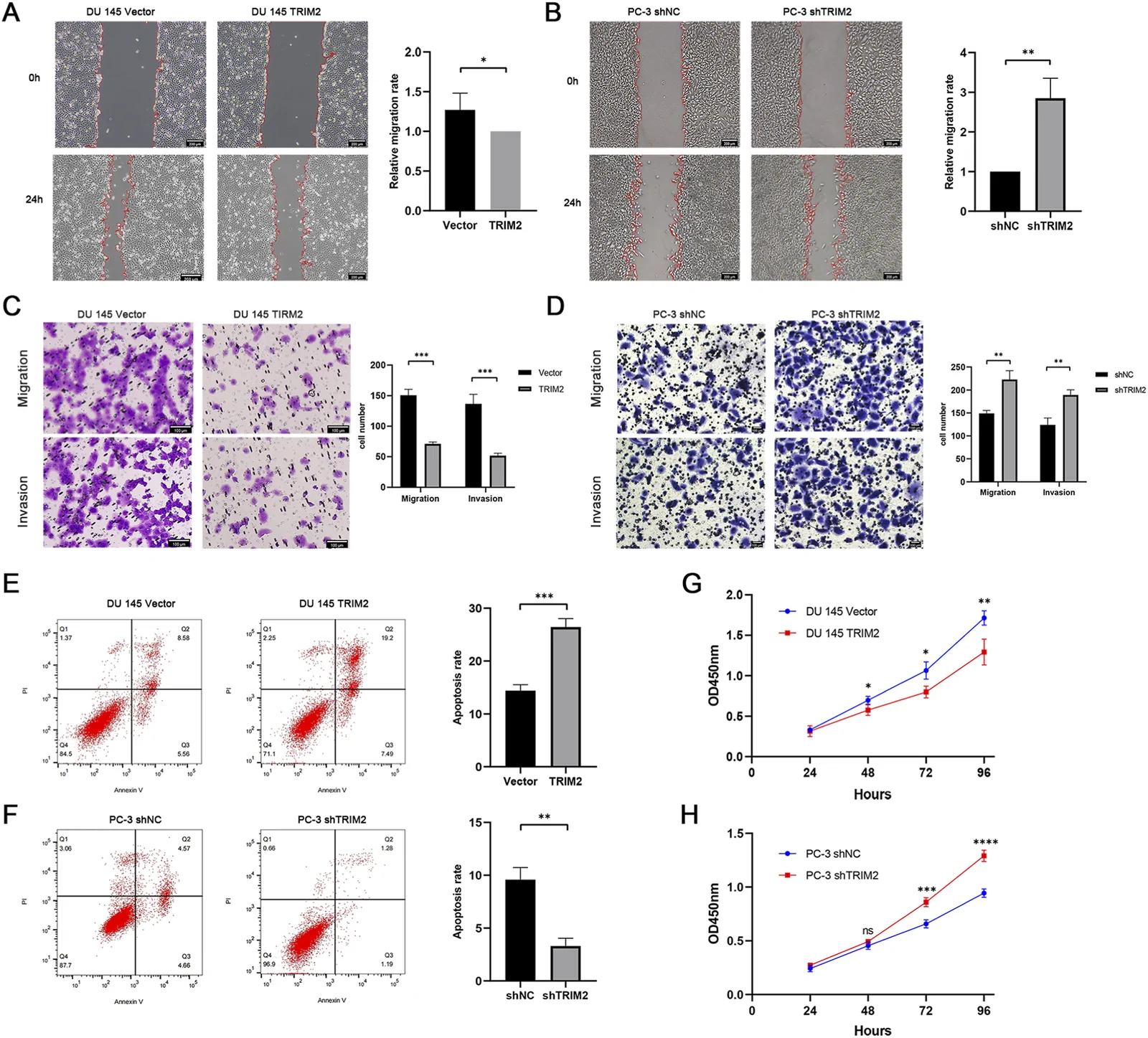
TRIM2 regulates malignant phenotypes of prostate cancer cells. **(A–B)** Migratory capacity of DU 145 cells overexpressing TRIM2 and PC-3 cells with TRIM2 knockdown was examined by wound healing assays. **(C–D)** Migration and invasion abilities were assessed via Transwell assays in the same cell lines. **(E–F)** Apoptosis rates of DU 145 cells upon TRIM2 overexpression and PC-3 cells upon TRIM2 knockdown were analyzed using flow cytometry **(G–H)** Cell proliferation was measured by CCK-8 assays. *P < 0.05, **P < 0.01, ***P < 0.001, ****P < 0.0001.

### 3.5 TRIM2 overexpression impairs subcutaneous tumor formation in nude mice

We conducted subcutaneous xenograft experiments in nude mice using DU 145 cells with stable TRIM2 overexpression or empty vector. Compared with the control group, TRIM2 overexpression led to significantly reduced tumor volume and weight (Figures 5A–C). Western blot analysis confirmed markedly higher TRIM2 protein levels in xenografts from TRIM2-overexpressing cells. Based on previous *in vitro* findings and relevant literature (Xu et al., 2021), TRIM2 may influence apoptosis by modulating apoptosis-related proteins. To validate this mechanism *in vivo*, the Bim and Caspase-3 expression levels were assessed. Notably, TRIM2 overexpression strongly upregulated both Bim and Caspase-3 in tumor tissues (Figure 5D). Subsequently, the tumor tissue’s pathological structure and cellular morphology were observed via HE staining (Figure 5E). IHC analysis further showed that TRIM2-overexpressing tumors exhibited markedly elevated TRIM2 levels, along with a notable reduction in the proliferation marker Ki67 (Figures 5F,G). The above experiments demonstrate that therapeutic restoration of TRIM2 activity could represent a promising strategy to impede prostate cancer progression and manage advanced disease.

**FIGURE 5:**
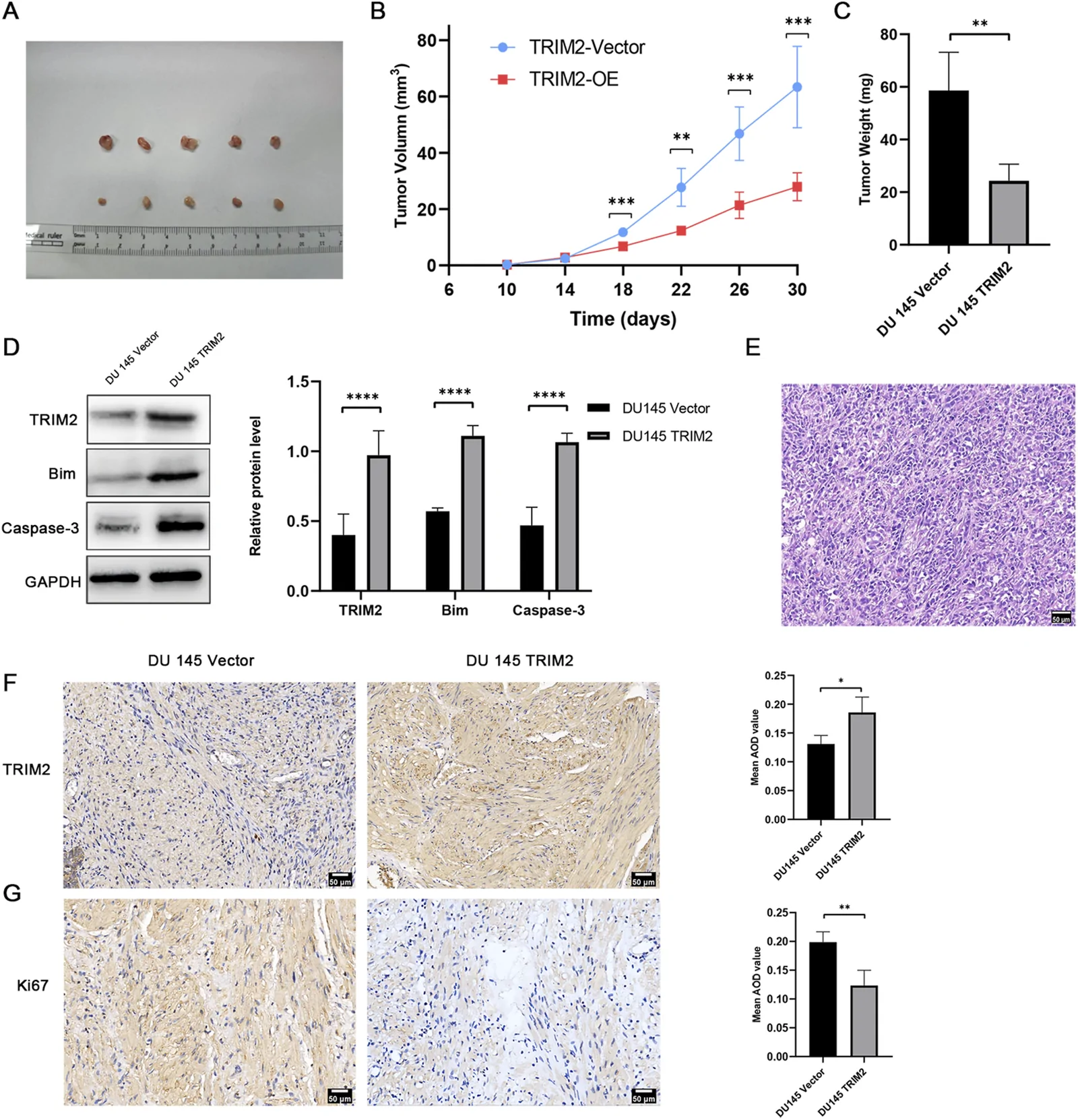
Effect of TRIM2 expression on tumorigenicity in nude mice. **(A-C)** Subcutaneous tumor formation capacity of DU 145 cells with TRIM2 overexpression versus control cells in nude mice.; **(D)** Apoptosis-associated proteins Bim and Caspase-3 were analyzed via Western blot in xenograft tissues. **(E)** HE staining showing the morphological features of xenograft tumor cells; **(F–G)** IHC showing TRIM2 and Ki-67 expression in xenograft tumors. *P < 0.05, **P < 0.01, ***P < 0.001, ****P < 0.0001.

### 3.6 Enrichment analysis of TRIM2 in PCa

We first screened for TRIM2-related DEGs, this yielded 1,352 upregulated and 49 downregulated genes (Figure 6A). Displayed in the heatmap are the top 50 upregulated genes and all 49 downregulated genes. (Figure 6B). GO analysis showed that TRIM2-associated genes were enriched in cell−cell signaling by wnt, Wnt signaling pathway, and cell junction assembly (BP); cell–cell junctions, collagen-containing extracellular matrix (CC) and actin/cadherin binding (MF) (Figure 6C). Significant enrichment of the PI3K-Akt pathway, human papillomavirus infection, and focal adhesion was revealed by KEGG (Figure 6D). Furthermore, GSEA demonstrated that the TRIM2 high-expression was significantly enriched in ubiquitin-mediated proteolysis, adherens junction, colorectal cancer, and spliceosome pathways within the KEGG gene sets (Figure 6E). In the Hallmark gene sets, significant enrichment was observed in protein secretion, UV response downregulated, and TGF-β signaling pathways (Figure 6F). Taken together, TRIM2 may drive PCa progression via the ubiquitin-proteasome pathway.

**FIGURE 6:**
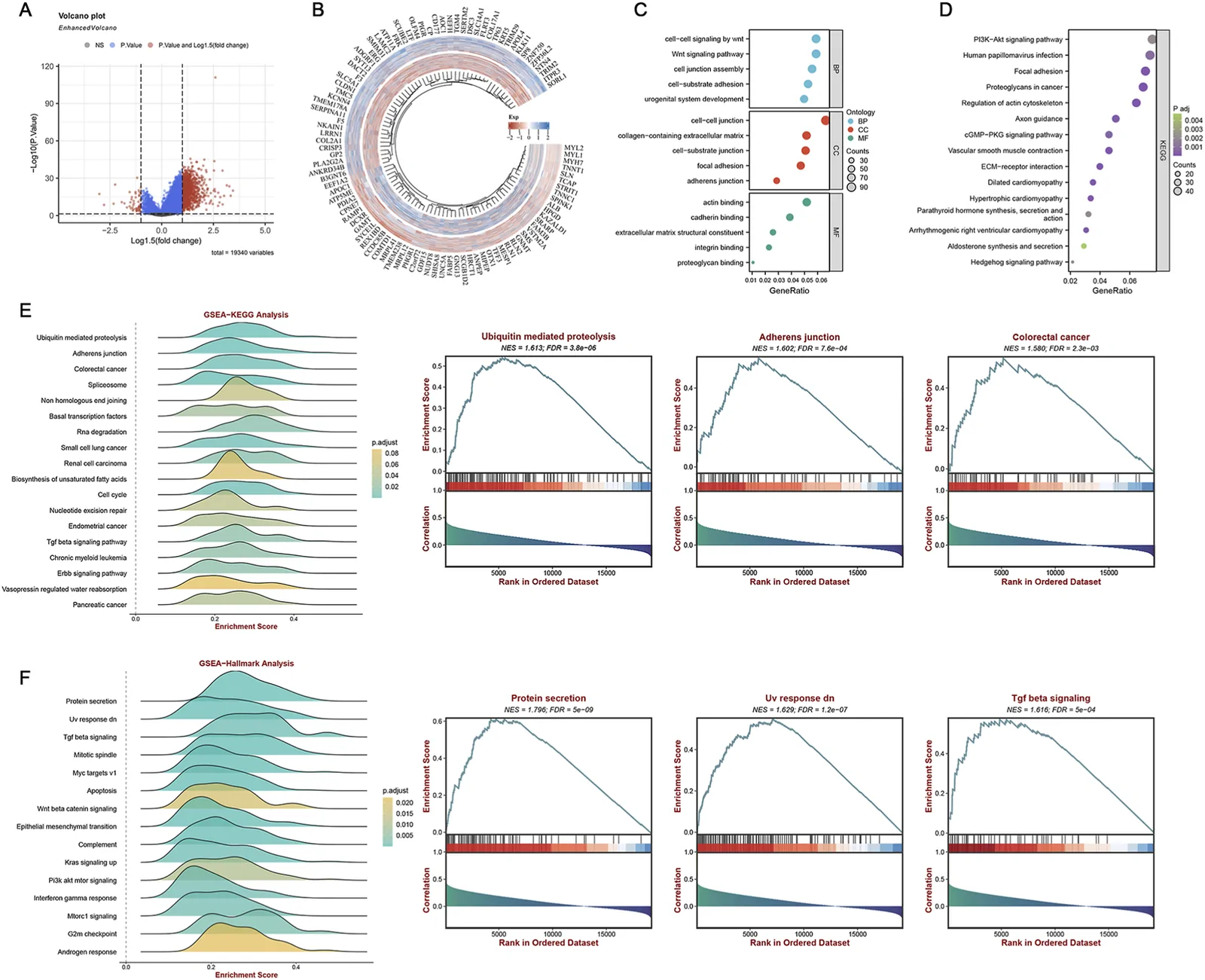
Pathway enrichment analyses of TRIM2 in prostate cancer. **(A)** Differential expression analysis of genes of TRIM2 in PCa. **(B)** The 50 most positively and 49 most negatively TRIM2-associated genes. **(C–D)** GO and KEGG enrichment results for TRIM2-related genes. **(E–F)** GSEA based on TRIM2 expression levels using the C2 cp. All gene set and the Hallmarks gene set, showing the top three most significantly enriched pathways ranked by the |NES|.

### 3.7 TRIM2 regulates DTNBP1 expression via the protein-ubiquitin-proteasome pathway

Using integrated analysis of proteomic sequencing data and predictions from UbiBrowser and BioGRID databases. We identified three potential ubiquitination substrates of TRIM2: Bim, TP53, and DTNBP1 (Dysbindin-1, dystrobrevin binding protein 1) (Figure 7A) (Supplementary Table S1). Given that the TRIM2–Bim interaction has been previously established (Thompson et al., 2011) and that TP53 is a well-studied tumor suppressor (Voskarides and Giannopoulou, 2023), we focused on DTNBP1, whose role in prostate cancer remains unexplored. According to TCGA data, DTNBP1 expression was markedly upregulated in PCa tissues (Figure 7B). Moreover, Proteomic sequencing data further showed an inverse relationship between TRIM2 and DTNBP1 expression (r = −0.917). Based on these findings, we hypothesized that TRIM2 functionally interacts with DTNBP1 and regulates its expression, thereby influencing prostate cancer progression.

**FIGURE 7:**
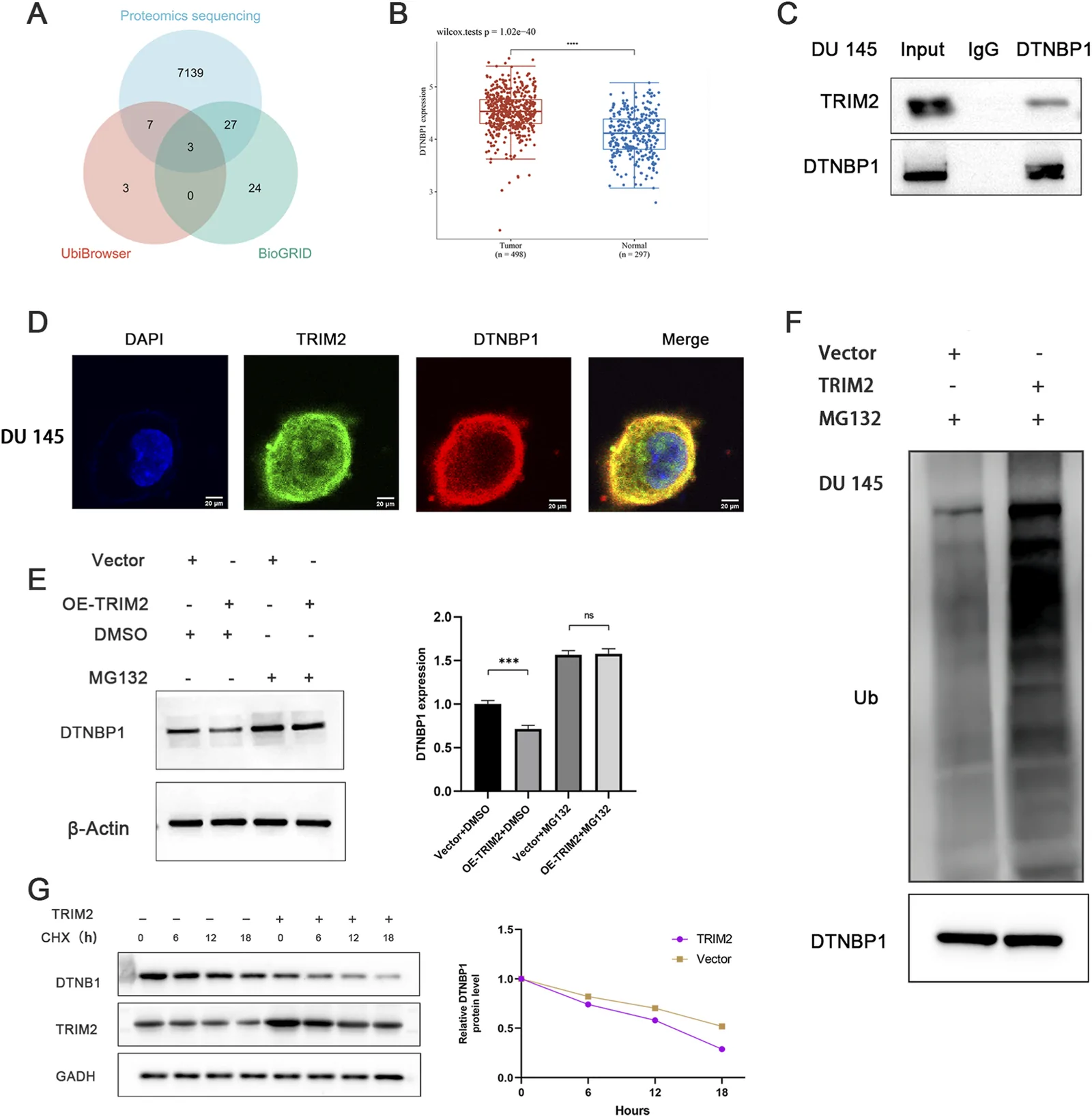
TRIM2 regulates DTNBP1 expression via the ubiquitin-proteasome pathway. **(A)** Identification of potential TRIM2-interacting proteins based on quantitative proteomic analysis and online database predictions. **(B)** Analysis of DTNBP1 expression levels in PCa tissues. **(C)** Co-IP validation of the TRIM2-DTNBP1 interaction. **(D)** Immunofluorescence showing the co-localization of TRIM2 with DTNBP1 in DU 145 cells. **(E)** Western blot analysis of the effect of the proteasome inhibitor MG132 on DTNBP1 protein levels in TRIM2-overexpressing DU 145 cells. **(F)** Ubiquitination assay measuring DTNBP1 ubiquitination levels upon TRIM2 overexpression in DU 145 cells. **(G)** DTNBP1 protein degradation in TRIM2-overexpressing DU 145 cells following cycloheximide (CHX) treatment at indicated time points. ***P < 0.001, ****P < 0.0001.

To further corroborate the TRIM2-DTNBP1 interaction, Co-IP assays were first performed, confirmed their interaction (Figures 7C,D). Furthermore, immunofluorescence staining showed colocalization of the two proteins in prostate cancer cells (Figure 7E). Based on the results of the GSEA analysis, we further investigated whether TRIM2 regulates DTNBP1 expression via the ubiquitin-proteasome pathway. The results showed that overexpressing TRIM2 significantly decreased DTNBP1 protein levels, an effect completely blocked by the proteasome inhibitor MG132 (Figure 7F). Under MG132 treatment, immunoprecipitation assays further showed that DTNBP1 ubiquitination was substantially enhanced in TRIM2-overexpressing DU 145 cells compared with controls (Figure 7G). Moreover, CHX chase assays indicated that TRIM2 overexpression significantly accelerated the degradation rate of DTNBP1 (Figure 7H). Collectively, these results demonstrate that TRIM2 downregulates DTNBP1 at the post-translational level by facilitating its ubiquitination and proteasomal degradation.

### 3.8 Correlation between TRIM2 expression and sensitivity to antitumor agents

We explored the impact of TRIM2 expression in altering the sensitivity of prostate cancer cells to clinically relevant drugs by measuring the IC50 values for four representative agents: olaparib (PARP inhibitor), bicalutamide (antiandrogen), docetaxel (chemotherapeutic), and navitoclax (BCL-2 inhibitor). High TRIM2 expression was significantly and positively correlated with increased IC50 values for bicalutamide and docetaxel (Figures 8A,B), suggesting a potential association with reduced sensitivity to these two agents *in silico*. Conversely, low TRIM2 expression was significantly negatively correlated with increased IC50 values for olaparib and navitoclax (Figures 8A,B), indicating a potential association with increased sensitive to these two agents, which warrants further experimental investigation.

**FIGURE 8:**
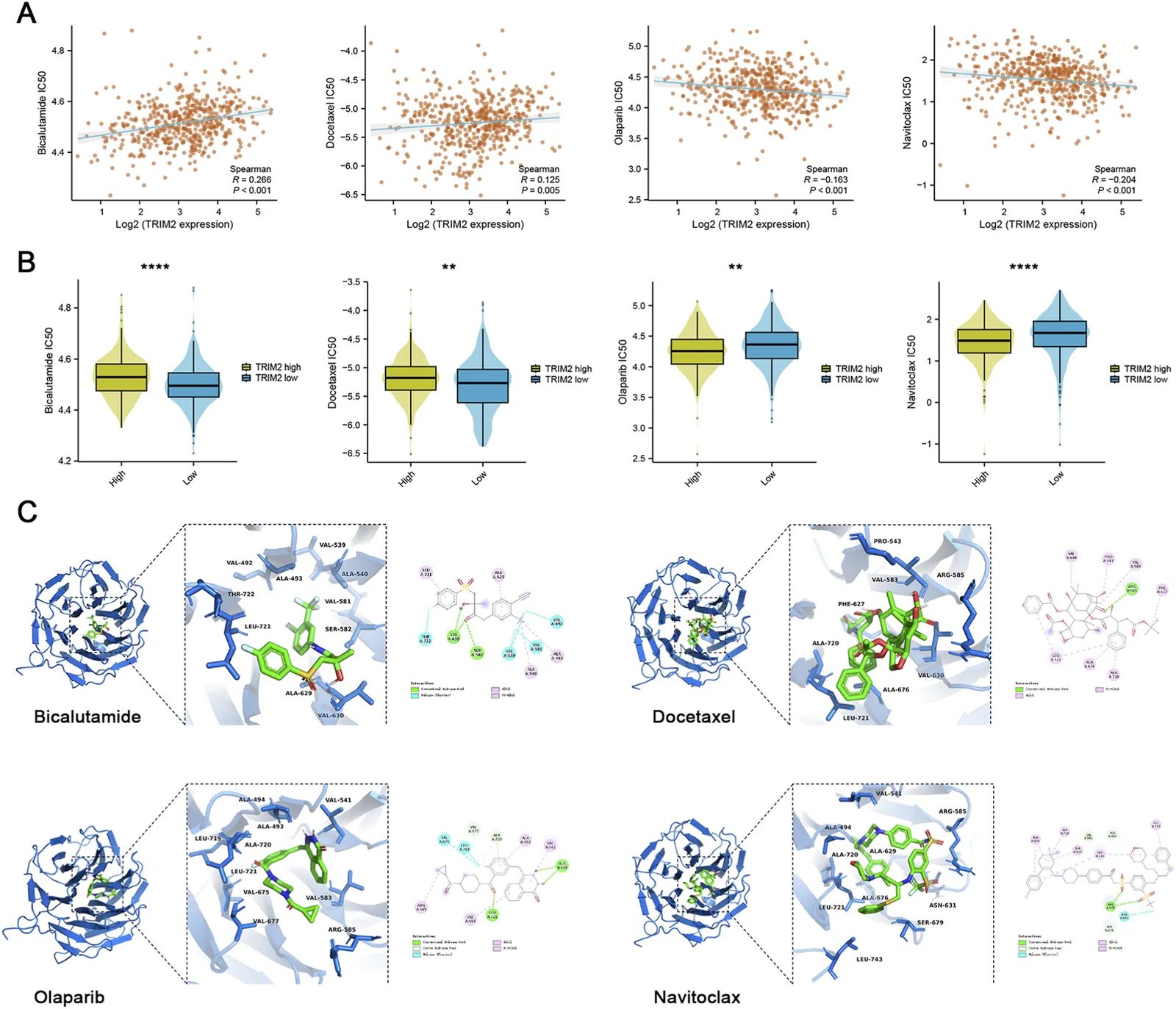
Correlation analysis of TRIM2 expression with drug sensitivity and molecular docking of TRIM2 with anti-tumor agents. **(A)** Relationship between TRIM2 expression and the IC50 of bicalutamide, docetaxel, olaparib, and Navitoclax. **(B)** Variations in IC50 values for the four drugs observed in PCa characterized by high or low TRIM2 expression. **(C)** Schematic diagrams illustrating the molecular docking of TRIM2 with the four compounds. **P < 0.01, ****P < 0.0001.

We next evaluated TRIM2 binding to four compounds via molecular docking. The binding energies of olaparib, docetaxel, and navitoclax with TRIM2 were −8.945, −8.793, and −8.954 kcal/mol, with corresponding dissociation constants (KD) of 0.275, 0.355, and 0.271, respectively, all indicative of strong binding affinity. Meanwhile, bicalutamide exhibited a lower binding energy (−7.606 kcal/mol) and a KD of 2.637, suggesting weaker interaction (Figure 8C). These findings noted that high TRIM2 expression status could serve as a valuable predictive biomarker for patient stratification, particularly in identifying candidates who are most likely to benefit from PARP or BCL-2 inhibitor-based regimens in the setting of advanced or CRPC patients.

## 4 Discussion

Prostate cancer, a common malignancy derived from glandular epithelial cells, represents the most prevalent malignancy of the male genitourinary tract and ranks second among cancer-related causes of death in men (Siegel et al., 2023). The disease has shown persistently rising incidence and death rates (Ju et al., 2023). Although localized PCa can be effectively managed with surgery or radiotherapy, treatment options for advanced PCa remain limited. Hence, optimizing early detection methods and molecular subtyping systems, along with clarifying the underlying molecular mechanisms driving PCa progression, will provide a critical theoretical basis for developing biomarker-guided personalized therapies and enhancing patient outcomes.

The TRIM protein family, a medium-sized subfamily comprising over 80 members, is primarily involved in post-translational modifications, with the majority exhibiting E3 ubiquitin ligase activity (Ozato et al., 2008; Esposito et al., 2017). TRIM2 also possesses this enzymatic function. Prior investigations have demonstrated that TRIM2 influences Snail1 expression via deubiquitination, thereby influencing the progression of lung adenocarcinoma (Lin et al., 2020). Furthermore, TRIM2 has been reported to facilitate clear cell renal cell carcinoma (ccRCC) progression by mediating ubiquitin-dependent proteolysis and may represent an independent prognostic marker for this malignancy (Wei et al., 2020). However, its expression profile and biological function in PCa remain largely unexplored. Therefore, we integrated bioinformatic analyses with experiments to systematically explore the functional relevance of TRIM2 in PCa.

Our analysis revealed that TRIM2 expression progressively declined during prostate cancer progression, reaching its lowest level in advanced stages and CRPC. Experimental validation subsequently verified reduced TRIM2 expression in PCa, but the relatively modest size of our in-house clinical validation cohort constitutes a limiting factor. In ROC analysis, TRIM2 exhibits excellent performance in discriminating localized from advanced/CRPC disease, suggesting its adjunctive value in clinical staging via biopsy-based assessment. Extremely low TRIM2 expression (under the optimal cutoff) signals increased susceptibility to aggressive progression, favoring intensified monitoring or early therapeutic intervention over active surveillance. Survival analyses demonstrated that TRIM2 was an independent predictor for PFI. Meanwhile, *in vitro* evidence indicates that reduced TRIM2 expression strongly augments the malignant behavior of cancer cells, raising the possibility that TRIM2 levels are further diminished in advanced prostate cancer, which indicates that TRIM2 expression status may serve as a valuable predictor for the risk of progression to advanced disease. However, large-scale, multicenter, prospective cohorts with well-annotated longitudinal follow-up are urgently required to validate the translational utility of TRIM2 as a routine clinical biomarker before its implementation in practice.

TRIM2 upregulation significantly attenuates, whereas its downregulation promotes the malignant biological behaviors of prostate cancer cells. GSEA -KEGG analysis revealed that high TRIM2 expression was strongly associated with adherens junction and epithelial-mesenchymal transition (EMT) signaling pathways. Epithelial polarity and tissue integrity depend critically on functional adherens junctions (Riga et al., 2020); loss of E-cadherin, a core adherens junction component, is recognized as a rate-limiting step and hallmark of EMT initiation (Thiery et al., 2009). EMT induces loss of epithelial polarity and adherens junction function, thereby facilitating wound healing, fibrosis, and cancer progression (Lamouille et al., 2014; Le Bras et al., 2012). Here, the positive association between TRIM2 expression and adherens junction pathway enrichment suggests that TRIM2 may stabilize the E-cadherin complex through direct or indirect mechanisms, thereby reinforcing cell–cell adhesion. This finding aligns with the evidence that TRIM2 overexpression reduces cell migration and invasion. Notably, TRIM2 high expression was also enriched in the EMT pathway when analyzed using the Hallmark gene set collection, which appears contradictory to the adherens junction enrichment. We speculate that this discrepancy may arise from the use of different MSigDB datasets for enrichment analysis. Previous studies have reported that TRIM2, in coordination with Snail, regulates EMT and consequently promotes tumor progression and metastasis in lung adenocarcinoma (Lin et al., 2020), and also control metastasis in colorectal cancer through EMT regulation (Cao et al., 2019). Therefore, whether TRIM2 modulates prostate cancer progression through adherens junction or EMT signaling pathways warrants further experimental studies.

Caspase-3, a central protease in the apoptotic pathway, has its activation widely considered an irreversible hallmark of apoptosis, making it a reliable biomarker for detecting apoptotic cells (Asadi et al., 2022). Bim, a BH3-only protein, interacts with all anti-apoptotic Bcl-2 family members with high affinity, which initiates the intrinsic apoptotic pathway under different circumstances (Edlich, 2018; Shukla et al., 2017). Our flow cytometry analysis revealed that TRIM2 overexpression significantly promoted apoptosis, whereas TRIM2 knockdown markedly suppressed it. These findings were further corroborated by *in vivo* subcutaneous xenograft assays, in which TRIM2 overexpression not only inhibited tumor growth but also upregulated Bim and Caspase-3 expression. Notably, elevated TRIM2 expression in PCa patients was found to confer higher sensitivity to Navitoclax—a BH3-mimetic agent that selectively inhibits anti-apoptotic Bcl-2 family members via competitive binding, thereby neutralizing their suppression of apoptotic signaling (Delbridge and Strasser, 2015; Nor Hisam et al., 2021; Tse et al., 2008). This suggests that elevated TRIM2 expression may potentiate the anti-tumor efficacy of BH3 mimetics like Navitoclax, potentially through the upregulation of pro-apoptotic mediators such as Bim. Furthermore, correlation analysis suggests that patients with high TRIM2 expression exhibited increased sensitivity to olaparib. Given that PARP inhibitors confer clinical benefit predominantly in prostate cancers with homologous recombination repair (HRR) deficiencies (de Bono et al., 2020; Hussa et al., 2020), our finding raises the possibility that TRIM2 expression may be associated with an HRR-deficient phenotype or with enhanced replication stress. Collectively, these results establish TRIM2 as both a promising companion biomarker for patients who may benefit from PARP or BCL-2 inhibitors and a potential therapeutic target in advanced PCa. Nevertheless, the GDSC-based predictions and molecular docking analyses are purely exploratory and lack direct biochemical evidence of binding. Biophysical validation is indispensable to confirm direct interactions, and prospective clinical cohorts are urgently needed to validate the translational potential of our findings.

To identify potential ubiquitination substrates of TRIM2, we performed proteomic sequencing combined with database prediction and identified DTNBP1 as a candidate target. DTNBP1, also known as BLOC1S8, is a core structural element of the biogenesis of lysosome-related organelles complex 1 (BLOC-1), contributing to organelle biogenesis (Ghiani and Dell'Angelica, 2011). Initially characterized as a dystrophin-associated protein, DTNBP1 serves as a key subunit of the dystrophin-associated glycoprotein complex (DPC), playing essential roles in maintaining sarcolemmal stability and signal transduction (Benson et al., 2001). In addition, DTNBP1 regulates multiple biological processes, including protein trafficking, transcriptional regulation, neurite extension, and synaptic vesicle formation (Ghiani and Dell'Angelica, 2011; Mullin et al., 2011). Accumulating studies have established a pivotal role for DTNBP1 in schizophrenia, showing markedly reduced expression levels in patients’ hippocampus, prefrontal cortex, and auditory cortex (Talbot et al., 2011; Tang et al., 2009; Weickert et al., 2008). Moreover, its promoter DNA methylation status has even been proposed as a diagnostic biomarker (Abdolmaleky et al., 2015). In cancer biology, however, the role of DTNBP1 remains underexplored. In pancreatic ductal adenocarcinoma, miR-342–3p directly targets DTNBP1, which in turn facilitates tumor progression through activating the NF-κB/MDM2 axis (Guo et al., 2016; Zhu et al., 2020). In hepatocellular carcinoma, DTNBP1 knockdown suppresses proliferation and metastatic potential, triggers apoptosis, and alters the cell cycle-related gene expression (Cheng et al., 2022). In brain tumor patients, DTNBP1 single nucleotide polymorphisms correlate strongly with cognitive function scores (Correa et al., 2016). Notably, two TRIM family members, TRIM24 and TRIM32, have been shown to regulate DTNBP1 expression through ubiquitination, thereby influencing cardiomyocyte survival, hypertrophy, and apoptosis (Borlepawar et al., 2017). These findings lead us to hypothesize that TRIM2 similarly ubiquitinates DTNBP1, thus promoting prostate cancer. But the direct causal relationship between the TRIM2-DTNBP1 axis and the observed tumor-suppressive phenotypes warrants further dissection. Specifically, whether DTNBP1 re-expression can effectively rescue the inhibitory effects induced by TRIM2 overexpression remains to be experimentally addressed.

As an essential post-translational modification, ubiquitination is defined as the covalent conjugation of ubiquitin molecules to protein substrates, typically leading to their proteasomal degradation (Dantuma and Masucci, 2002). Beyond proteolysis, ubiquitination also regulates non-proteolytic processes including transcription, translation, DNA damage repair, protein trafficking, and signal transduction (Dougherty et al., 2020; Hirayama et al., 2018; Wang et al., 2018). The dynamic equilibrium between ubiquitination and deubiquitination critically governs both tumor initiation and progression (Abbas and Dutta, 2017; Puhr et al., 2016). Our GSEA results showed significant enrichment of the “ubiquitin-mediated proteolysis” pathway in the TRIM2-high group, further supporting the notion that TRIM2 functions through ubiquitination. Integrating proteomic sequencing with public databases, we identified DTNBP1 as a candidate downstream target of TRIM2. Subsequent assays verified the TRIM2-DTNBP1 interaction and further demonstrated that TRIM2 facilitates the ubiquitin-proteasome-dependent degradation of DTNBP1. TRIM2 harbors E3 ubiquitin ligase activity via its RING domain (Balastik et al., 2008), while Coiled-Coil and B-BOX domains act cooperatively in substrate recognition and binding (Li et al., 2014; Micale et al., 2012; Xiao et al., 2022). Interestingly, DTNBP1 also contains a Coiled-Coil domain (Benson et al., 2001), a structural commonality that suggests a more refined regulatory mechanism. Whether DTNBP1 ubiquitination is directly catalyzed by the RING domain of TRIM2 or requires prior recruitment via Coiled-Coil domain-mediated interactions remains to be elucidated. Different ubiquitin chain linkages confer distinct biological functions on ubiquitinated proteins, K48 polyubiquitin modification preferentially facilitates proteasomal degradation, and K63-linked ubiquitination is responsible for non-degradative cellular events (Liu F. et al., 2024). In nasopharyngeal carcinoma, TRIM21 has been reported to degrade VDAC2 via K48-linked ubiquitination, thereby inhibiting cGAS/STING-mediated immune surveillance (Li et al., 2023). Our finding that DTNBP1 is degraded via the proteasome implies that K48-linked ubiquitination likely predominates. Nevertheless, the exact ubiquitin linkage type and the critical modification sites involved in TRIM2-mediated DTNBP1 ubiquitination in prostate cancer remain to be clarified, this constitutes a mechanistic limitation, and restoring TRIM2 activity or abrogating its downstream oncogenic substrate, DTNBP1, could represent a novel therapeutic strategy.

## 5 Conclusion

Our findings identify TRIM2 as a key tumor suppressor whose expression is further downregulated in advanced PCa, it demonstrates outstanding accuracy in distinguishing localized from advanced/CRPC cases. And low TRIM2 independently forecasts worse PFI. TRIM2 overexpression suppresses proliferation, migration, and invasion, while markedly inducing apoptosis. Mechanistically, TRIM2 directly targets DTNBP1 for degradation via the ubiquitin-proteasome pathway, thereby driving its tumor-suppressive effects. We also provide crucial evidence that TRIM2 expression correlates with the efficacy of clinically relevant targeted agents, notably PARP and BCL-2 inhibitors. Taken together, our observations confirm the oncogenic relevance of the TRIM2–DTNBP1 interaction in prostate cancer and highlight the translational potential of TRIM2 as a dual-purpose biomarker for prognostic assessment and therapeutic prediction.

## Data Availability

The data presented in the study are deposited in the Science Data Bank repository, with the DOI: 10.57760/sciencedb.011ew.
